# Advancing Prostate Cancer Diagnostics: A ConvNeXt Approach to Multi-Class Classification in Underrepresented Populations

**DOI:** 10.3390/bioengineering12040369

**Published:** 2025-04-01

**Authors:** Declan Ikechukwu Emegano, Mubarak Taiwo Mustapha, Ilker Ozsahin, Dilber Uzun Ozsahin, Berna Uzun

**Affiliations:** 1 Operational Research Centre in Healthcare, Near East University, TRNC Mersin 10, Nicosia 99138, Turkeydozsahin@sharjah.ac.ae (D.U.O.); berna.uzun@neu.edu.tr (B.U.); 2 Department of Medical Diagnostic Imaging, College of Health Sciences, University of Sharjah, Sharjah 27272, United Arab Emirates; 3 Research Institute of Medical and Health Sciences, University of Sharjah, Sharjah 27272, United Arab Emirates; 4 Department of Mathematics, Near East University, TRNC Mersin 10, Nicosia 99138, Turkey

**Keywords:** ConvNeXt, CNN, Grad-CAM, histopathological images, multi-class classification, prostate cancer, sub-Saharan Africa

## Abstract

Prostate cancer is a leading cause of cancer-related morbidity and mortality worldwide, with diagnostic challenges magnified in underrepresented regions like sub-Saharan Africa. This study introduces a novel application of ConvNeXt, an advanced convolutional neural network architecture, for multi-class classification of prostate histopathological images into normal, benign, and malignant categories. The dataset, sourced from a tertiary healthcare institution in Nigeria, represents a typically underserved African population, addressing critical disparities in global diagnostic research. We also used the ProstateX dataset (2017) from The Cancer Imaging Archive (TCIA) to validate our result. A comprehensive pipeline was developed, leveraging advanced data augmentation, Grad-CAM for interpretability, and an ablation study to enhance model optimization and robustness. The ConvNeXt model achieved an accuracy of 98%, surpassing the performance of traditional CNNs (ResNet50, 93%; EfficientNet, 94%; DenseNet, 92%) and transformer-based models (ViT, 88%; CaiT, 86%; Swin Transformer, 95%; RegNet, 94%). Also, using the ProstateX dataset, the ConvNeXt model recorded 87.2%, 85.7%, 86.4%, and 0.92 as accuracy, recall, F1 score, and AUC, respectively, as validation results. Its hybrid architecture combines the strengths of CNNs and transformers, enabling superior feature extraction. Grad-CAM visualizations further enhance explainability, bridging the gap between computational predictions and clinical trust. Ablation studies demonstrated the contributions of data augmentation, optimizer selection, and learning rate tuning to model performance, highlighting its robustness and adaptability for deployment in low-resource settings. This study advances equitable health care by addressing the lack of regional representation in diagnostic datasets and employing a clinically aligned three-class classification approach. Combining high performance, interpretability, and scalability, this work establishes a foundation for future research on diverse and underrepresented populations, fostering global inclusivity in cancer diagnostics.

## 1. Introduction

Prostate cancer is a significant global health concern, ranking among the most commonly diagnosed malignancies in men and a leading cause of cancer-related mortality [1,2,3]. However, the disease burden is not evenly distributed, with men of African descent—particularly those in sub-Saharan Africa—facing higher risks, more aggressive disease phenotypes, and poorer clinical outcomes compared to other populations [4,5,6]. Despite these disparities, prostate cancer research has primarily focused on Western populations, leading to diagnostic and therapeutic frameworks that may not fully account for the unique biological and demographic characteristics of African men [7]. Studies have emphasized the urgency of addressing these disparities, as African populations experience higher mortality rates due to aggressive disease presentation and limited diagnostic resources [8,9].

Artificial intelligence (AI) and deep learning have shown promise in improving diagnostic accuracy and workflow efficiency in prostate cancer detection [10]. While histopathological analysis remains the gold standard for diagnosis, its manual and subjective nature introduces challenges, particularly in resource-limited settings where specialized expertise is scarce [11,12]. These limitations contribute to diagnostic delays and inconsistencies. AI-driven approaches offer a transformative solution by enhancing the analysis of histopathological images, yet most existing models are limited to binary classification, restricting their clinical utility [13,14]. Furthermore, many AI studies rely on datasets from homogeneous, non-representative populations, overlooking demographic variations that may influence disease characteristics and progression [15].

Recent advancements in hybrid architectures, combining convolutional neural networks (CNNs) with transformers, have demonstrated strong performance in medical imaging tasks, including prostate cancer classification. Studies such as Zhang et al. (2022) [16] have highlighted the effectiveness of CNN–transformer hybrids in histopathological analysis, while Kumar et al. [10] stressed the importance of demographically diverse datasets to enhance AI model generalizability in oncology.

This study aims to address these gaps by leveraging ConvNeXt, an advanced neural network architecture that integrates the computational efficiency of CNNs with the contextual learning capabilities of transformers [17]. We propose a three-class classification framework to distinguish between normal, benign, and malignant prostate histopathological images. Our dataset, sourced from a Nigerian tertiary healthcare institution, provides a rare and valuable representation of African populations, helping to mitigate the chronic underrepresentation in AI-driven prostate cancer research [18].

Additionally, we incorporate Gradient-weighted Class Activation Mapping (Grad-CAM) to enhance interpretability, ensuring greater transparency and trust in clinical deployment [19,20]. Grad-CAM bridges the gap between AI predictions and human understanding by providing biologically meaningful visual explanations, an essential step towards integrating AI into routine diagnostics.

This study pioneers the application of ConvNeXt for three-class classification in prostate cancer histopathology, addressing regional disparities while advancing equitable AI applications in cancer diagnostics. By improving diagnostic accuracy, explainability, and clinical relevance, our work aims to contribute to more inclusive and effective prostate cancer detection strategies.

## 2. Related Studies

Prostate cancer is one of the most prevalent malignancies among men, accounting for a significant proportion of cancer-related morbidity and mortality worldwide. Accurate diagnosis relies heavily on histopathological examination of tissue biopsies, often assessed using the Gleason grading system. However, this manual diagnostic process is inherently subjective, with inter-observer variability being a longstanding issue, particularly in borderline cases. As healthcare systems move towards precision diagnostics, there is an increasing demand for computational approaches to enhance accuracy, reproducibility, and efficiency in prostate cancer diagnosis [21,22].

AI has transformed medical imaging, enabling unprecedented advancements in histopathological analysis. Convolutional neural networks (CNNs) have become a dominant paradigm due to their ability to learn and extract hierarchical features automatically. Studies have demonstrated their utility in the binary classification of prostate cancer histopathological images, achieving high sensitivity and specificity [23,24]. However, traditional CNNs often struggle with capturing global context and long-range dependencies, limiting their performance on complex tissue structures.

To address CNNs’ limitations, researchers have integrated transformer architectures into image analysis workflows. Initially developed for natural language processing tasks, transformers utilize self-attention mechanisms to model long-range dependencies, proving highly effective in medical imaging contexts. Vision Transformer (ViT) has been successfully applied to various imaging modalities, including histopathological image analysis, showcasing its ability to outperform traditional CNNs in capturing intricate patterns [25,26]. Despite their promise, transformer models generally require large datasets, posing challenges for medical applications where annotated data is often scarce.

Introduced in 2022, ConvNeXt represents a milestone in neural network development. By rethinking traditional CNNs through the lens of transformer design principles—such as using larger kernel sizes and inverted bottleneck layers—ConvNeXt achieves a balance between efficiency and performance. It retains the advantages of convolutional operations while enhancing its ability to capture global dependencies [27]. This hybrid architecture offers a unique opportunity for histopathological image analysis, particularly in prostate cancer detection, where both local and global image features are critical.

Additionally, drawing on an extensive range of sources, the authors set out how prostate cancer is diagnosed using diverse CNN architecture. Tsehay et al. [28] designed a CNN-based computer framework that could identify prostate cancer on multiparametric magnetic resonance images (mpMRI) to make it easier for readers to reach a consensus on what they see as well as improve the algorithm sensitivity. However, Sobecki et al. [29] presented an innovative influence of domain expertise encoding inside CNN model architecture for prostate cancer diagnosis utilizing Multiparametric MRI (mpMRI) images and executed late (decision-level) fusion for mpMRI data, employing distinct networks for each mp series. As a result, the precision of prostate cancer diagnosis was improved by utilizing specialized CNN architectures. Moreover, in her interesting recent study, Pirzad Mashak et al. [30] analyzed a novel methodology for identifying prostate cancer using magnetic resonance imaging (MRI) through the integration of both faster region-based convolutional neural networks (R-CNN) and CNN structures. A faster R-CNN was used for identification than a CNN-based network for data classification. The result showed enhanced accuracy in detecting prostate cancer using MRI images. On top of that, Rundo et al. [31] presented an innovative CNN framework, termed USE-Net, which integrated Squeeze and Excitation (SE) blocks into U-Net for zonal segmentation of the prostate using MRI images. This novel methodology underscores the significance of utilizing CNN frameworks for precise segmentation, especially in prostate cancer studies. Furthermore, Pinckaers et al. [32] developed a streamed implementation technique of CNN layers to train a contemporary CNN using image-level labeling for prostate cancer identification in a stained slide. This demonstrates the efficacy of data training throughout its entirety without requiring manually pixel-wise annotated images.

Most AI-driven studies on prostate cancer diagnosis have focused on binary classification, differentiating malignant and benign lesions. While effective, this approach overlooks the diagnostic value of incorporating “normal” tissues as a separate class, which can better align with clinical workflows. Few studies have ventured into three-class classification (normal, benign, malignant) despite its potential to improve diagnostic accuracy and clinical decision-making [33,34]. Additionally, existing datasets predominantly feature images from Western populations, limiting the generalizability of these models. Histological data from African or Black populations remain underrepresented despite evidence suggesting demographic differences in prostate cancer histopathology [35,36]. This lack of diversity underscores the need for inclusive datasets to develop robust AI systems applicable to global populations.

Beyond achieving high accuracy, interpretability is essential for deploying AI in healthcare settings. Gradient-weighted Class Activation Mapping (Grad-CAM) has emerged as a widely used method for visualizing model decisions by highlighting image regions that contribute to specific predictions. In prostate cancer diagnostics, Grad-CAM provides insights into how models interpret histopathological features, aiding clinicians in validating predictions and identifying potential errors [19]. This level of explainability fosters trust in AI models while ensuring alignment with clinical standards.

Ablation studies are pivotal in understanding model robustness by systematically modifying components or configurations. Altering data augmentation strategies, learning rate schedules, or optimizer types can reveal a model’s dependencies and sensitivities. These studies provide invaluable insights into the ConvNeXt architecture’s adaptability, enabling further optimization for histopathological applications [37,38].

## 3. Materials and Methods

### 3.1. Workflow of the Proposed Approach

**Figure 1:**
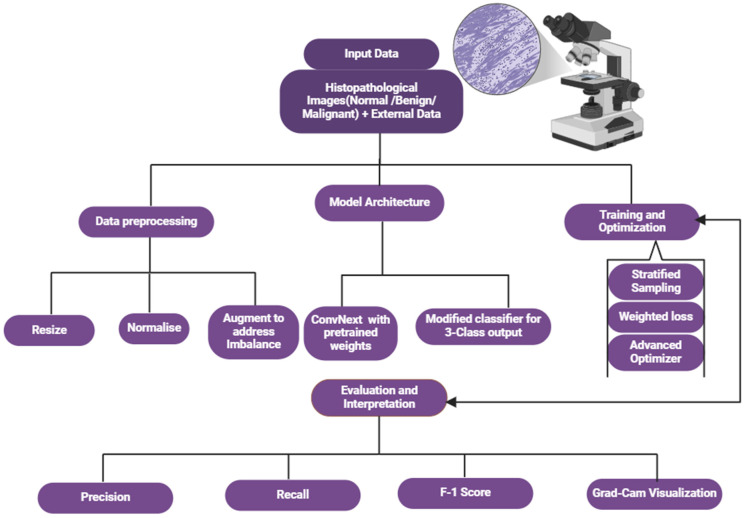
The workflow of the proposed approach.

Figure 1 outlines the various steps in conducting our experiments, including obtaining histopathological data, preprocessing the data, designing the model, training and optimizing it, evaluating it, and interpreting it.

### 3.2. Dataset

The dataset used in this study was collected from the Federal Medical Center Lokoja, Kogi State, Nigeria, over five years (2019–2024) from people between the ages of 39 and 80 years. The dataset used in this study was uniquely sourced from a tertiary healthcare institution in Nigeria. These data are particularly significant as they represent an underrepresented demographic with distinct biological and histopathological features of prostate cancer. While the dataset provides a valuable benchmark for model evaluation, we acknowledge that the reliance on a single-center dataset limits its broader applicability. However, its focus on an African population addresses a critical gap in global prostate cancer research, where such datasets are typically underrepresented.

**Figure 2:**
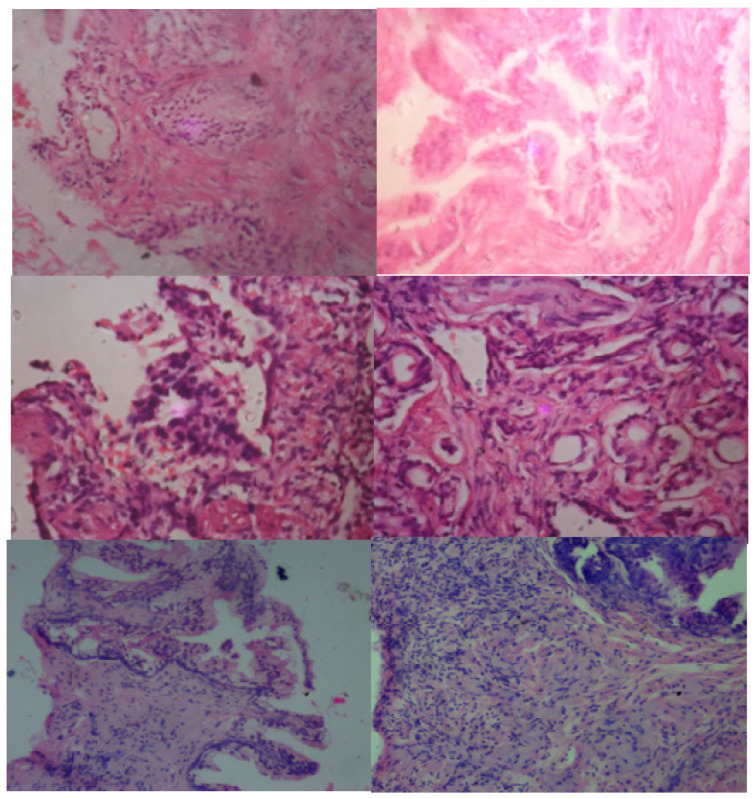
Prostate data (benign/malignant/normal).

Prostate tissue samples underwent hematoxylin and eosin (H&E) staining to reveal cellular and architectural features. Pathologists classified the samples into three categories—normal, benign, and malignant—based on established histopathological guidelines, including glandular structure and nuclear morphology. The dataset consisted of 1002 histological images, distributed as follows: 247 normal, 513 benign, and 242 malignant samples. This balance ensured that each class was equally represented during model training and evaluation. Ethical approval for this study was obtained from the Ethical Review Board of the Federal Medical Center, Lokoja (Approval Number: FMCL/HREC/Vol.I/2023/192). All patient data were anonymized to ensure confidentiality and compliance with international ethical standards. This dataset (Figure 2) provides a unique representation of prostate cancer in Nigerian and broader African populations, addressing the underrepresentation of these demographics in AI research. Ethical approval for this study was obtained from Federal Teaching Hospital Lokoja, Kogi State, Nigeria, adhering to strict patient confidentiality and ethical data usage standards.

### 3.3. Data Preprocessing

#### 3.3.1. Image Resizing and Standardization

All images were resized to 384 × 384 pixels to maintain uniform input dimensions suitable for model training. To standardize input features, pixel intensity values were normalized using ImageNet statistics ([0.485, 0.456, 0.406] mean and [0.229, 0.224, 0.225] standard deviation).

#### 3.3.2. Advanced Data Augmentation Techniques

To enhance model generalization and mitigate overfitting, a suite of data augmentation techniques was applied:

- Random Rotations: Rotations within ±15° accounted for orientation variability in histopathological samples.
- Horizontal and Vertical Flipping: A 50% probability of flipping simulated sample preparation and orientation variations.
- Color Jittering: Brightness, contrast, and saturation adjustments of ±10% mimicked staining variability in histopathology slides.
- Gaussian Noise: Random noise was added to simulate image artifacts and variability in slide digitization.

These techniques expanded the dataset, enabling the model to learn invariance to non-diagnostic variations. Augmentation parameters were optimized based on validation performance to avoid introducing distortions that could negatively impact model learning.

#### 3.3.3. Targeted Data Augmentation for Class Imbalance

To address the class imbalance, targeted augmentations were applied to malignant samples:

- Techniques such as rotations, flipping, and random cropping enhanced the diversity of malignant samples during training.
- A class-weighted cross-entropy loss function was employed, with weights inversely proportional to class frequencies. This ensured that misclassifications in underrepresented classes were penalized more heavily.
- Stratified batch sampling was implemented to maintain a proportional representation of each class within training batches, ensuring uniform learning across all classes.

#### 3.3.4. Dataset Splitting

The dataset was divided into training (70%), validation (15%), and test (15%) sets using stratified sampling to ensure balanced class representation across subsets. The StratifiedShuffleSplit module in scikit-learn was used to maintain proportional class distribution.

#### 3.3.5. Quality Control Measures

To ensure dataset integrity and reliable model training, several quality control measures were implemented:

- Visual Inspection: Two experienced pathologists independently reviewed all images to confirm diagnostic quality. Images deemed unclear, poorly stained, or blurred were excluded.
- Color Consistency: Histogram equalization was applied to mitigate variability in staining intensity, ensuring consistent color representation.
- Outlier Removal: Images with atypical characteristics (e.g., artifacts or background noise) were flagged and excluded.

### 3.4. Model Architecture

#### 3.4.1. Overview of ConvNeXt

**Figure 3:**
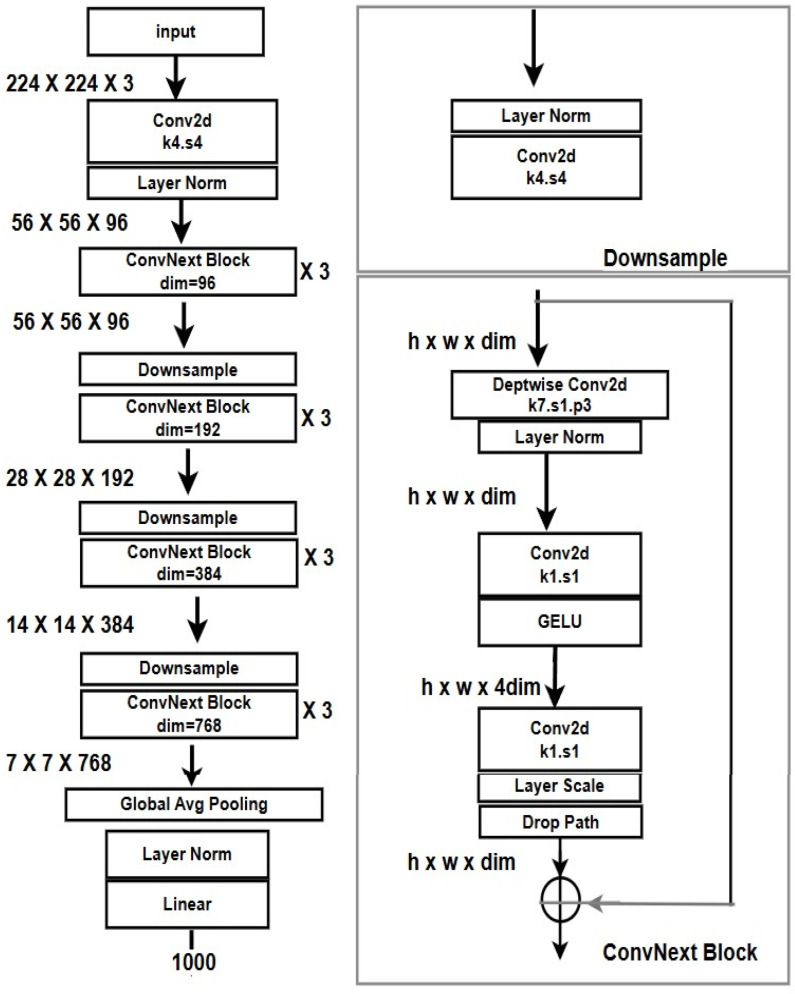
Architecture overview of ConvNeXT [40].

ConvNeXt, introduced by Liu et al. in 2022 [18], represents a major evolution in convolutional neural network (CNN) design, merging the efficiency of traditional CNNs with the performance benefits of Vision Transformers (ViTs). The model was developed to bridge the gap between these architectures, achieving state-of-the-art results on benchmark tasks like ImageNet classification while maintaining computational efficiency [27,39]. By refining the ResNet-inspired CNN paradigm with modern innovations such as larger kernels, simplified blocks, and GELU activations, ConvNeXt offers a streamlined yet powerful solution for complex visual tasks [27,38]. Its relevance to prostate cancer diagnosis lies in its ability to learn nuanced features in histopathological data while being computationally efficient enough for real-world clinical applications. Figure 3 shows the architecture overview of the ConvNeXT model.

In this study, we utilized ConvNeXt, a novel deep learning architecture that incorporates several important design choices, to improve the performance of convolutional neural networks (CNNs) in image recognition tasks, such as prostate cancer classification in mpMRI scans.

ConvNeXt builds upon the principles of ResNet but introduces several architectural modifications that enhance its ability to extract complex features from medical imaging data. In particular, ConvNeXt emphasizes the use of depthwise convolutions and large kernel sizes, which significantly improve feature extraction at various scales and levels of abstraction. Depthwise convolutions, as introduced in MobileNet architectures, reduce computational complexity while preserving spatial information. This modification allows the model to focus on local features and capture detailed information within the regions of interest in medical images, such as prostate lesions in mpMRI scans. Additionally, adopting large kernel sizes allows ConvNeXt to aggregate a broader context of the image in each convolution, helping the model better understand spatial relationships and improve classification accuracy, especially in complex visual data.

ConvNeXt also benefits from a CNN–transformer hybrid structure, which provides the flexibility to capture both local and global features in the image. While traditional CNNs excel at local feature extraction, transformers better capture long-range dependencies and contextual information. Combining these two components allows ConvNeXt to leverage the strengths of both CNNs and transformers, making it particularly suitable for medical image analysis, where fine-grained details and broader contextual understanding are essential for accurate diagnosis.

Furthermore, ConvNeXt’s architecture incorporates several design elements that help reduce overfitting and improve generalization. These include regularization techniques, such as dropout and batch normalization, and data augmentation strategies, which are crucial when working with limited medical datasets. These improvements collectively contribute to ConvNeXt’s superior performance in prostate cancer classification, as demonstrated by its higher accuracy, recall, F1 score, and AUC compared to traditional CNN models (e.g., ResNet50, DenseNet) and transformer-based architectures (e.g., ViT).

#### 3.4.2. Components of ConvNeXt

Key architectural innovations in ConvNeXt are summarized as shown in Table 1 and they include:

- Depthwise Separable Convolutions: Reduce computational complexity while preserving performance.
- Large Kernel Sizes: Use 7 × 7 kernels to capture richer spatial details.
- Normalization Layers: Employ layer normalization for training stability and compatibility with modern architectures.
- Linear Activation Functions: Replace ReLU with GELU for better pattern learning.
- Efficient Block Design: Simplify convolutional blocks by reducing the number of activation and normalization layers.
- Downsampling Layers: Use depthwise convolutions for downsampling, avoiding pooling operations to retain spatial continuity.

#### 3.4.3. Modifications for Three-Class Classification

The ConvNeXt model architecture, as adapted for this study, is summarized in Table 2. This architecture builds upon the ConvNeXt-Base variant, leveraging depthwise separable convolutions and large kernel sizes for efficient spatial feature extraction.

#### 3.4.4. Detailed Model Structure

The ConvNeXt model was adapted for three-class classification by replacing the default classifier head with a fully connected layer featuring three output neurons. A softmax activation function was applied to generate class probabilities. Pre-trained weights from ImageNet (IMAGENET1K_V1) were utilized for faster convergence and superior feature extraction, aligning the model with the specific requirements of histopathological analysis.

### 3.5. Grad-CAM for Explainability

Grad-CAM visualizations were generated using the PyTorch library version 2.6.0 with hooks implemented on the final convolutional layers of the ConvNeXt model. These visualizations highlighted the histologically relevant regions that contributed most to the model’s predictions [37,41]. This technique is particularly valuable in histopathology, where understanding the model’s focus helps validate predictions against histological expertise, fostering clinical trust and potential adoption.

### 3.6. Training Setup

The training process incorporated the following:

- Loss Function: Cross-entropy loss to manage the multi-class classification task.
- Optimizer: AdamW, initialized with a learning rate 1 × 10^−4^ for efficient weight updates.
- Learning Rate Scheduler: GradualWarmupLR stabilized learning for the initial five epochs, transitioning to CosineAnnealingLR for fine-tuning in subsequent epochs.
- Training Parameters: A batch size 64 was used for 30 epochs on an NVIDIA GPU to leverage accelerated computation and optimize model performance.

### 3.7. Evaluation Metrics

The performance of the ConvNeXt model was evaluated using several standard classification metrics: precision, recall, F1 score, accuracy, the area under the receiver operating characteristic curve (ROC-AUC), and the confusion matrix. These metrics were chosen for their ability to capture model performance in multi-class classification tasks comprehensively:

- Precision: This represents the proportion of true positive predictions out of all positive predictions for each class. This metric is crucial in reducing false positives, especially in medical diagnostics, where over-diagnosing a malignant case can lead to unnecessary interventions.
- Recall: This measures the proportion of true positives correctly identified by the model. High recall ensures minimal false negatives, which is particularly critical in identifying malignant cases.
- F1 Score: The harmonic mean of precision and recall, balancing the trade-off between false positives and false negatives. This metric is significant in assessing the overall reliability of the model in clinical scenarios.
- Accuracy: The proportion of correctly classified samples across all classes, offering a general measure of model performance.
- ROC-AUC: This evaluates the model’s ability to distinguish between classes by analyzing the trade-off between sensitivity and specificity. This study calculated the ROC-AUC using a one-versus-rest approach for multi-class classification.
- Confusion Matrix: This matrix provides a detailed breakdown of the model’s predictions by presenting the counts of true positives (TP), true negatives (TN), false positives (FP), and false negatives (FN) for each class. It allows for identifying specific areas of misclassification, enabling deeper insights into the model’s strengths and weaknesses.

These metrics were computed for each class (normal, benign, malignant) and averaged for overall performance. The confusion matrix, in particular, was instrumental in identifying subtle misclassification patterns, such as distinguishing benign from malignant cases. By combining these metrics, the study ensured a robust and clinically meaningful assessment of model performance.

### 3.8. Ablation Study

The ablation study examined the effects of data augmentation, optimization techniques, normalization, and learning rate configurations, as shown in Table 3. These configurations were selected to evaluate the robustness of the preprocessing pipeline and optimization strategies, which are critical for handling the variability in histopathological data.

### 3.9. Comparative Analysis with Existing Approaches

ConvNeXt’s hybrid architecture combines the strengths of convolutional neural networks (CNNs) and transformers, enabling it to extract intricate histopathological features while maintaining computational efficiency. This capability allows the model to generalize effectively, even in datasets with high heterogeneity, such as those representing underrepresented populations. Compared to approaches like ResNet50, EfficientNet, InceptionV3, ViT, CaiT, Swin Transformer, DenseNet, and RegNet, ConvNeXt provides superior performance in accuracy, recall, and F1 scores, as detailed in Table 4. Additionally, its explainability features, enhanced by Grad-CAM, address a critical need for interpretability in clinical applications, further distinguishing it from prior methods.

## 4. Results

### 4.1. Classification Performance

The ConvNeXt model demonstrated exceptional performance in classifying prostate cancer histopathological images into normal, benign, and malignant categories. Its key performance metrics, including precision, recall, F1 scores, and accuracy, underscore its robustness and reliability, as shown in Table 5.

These metrics were calculated on the test set for each class. Precision was determined as the ratio of true positives to the sum of true and false positives for each class. Recall measured the proportion of true positives correctly identified out of all actual positives. F1 score balanced the trade-offs between precision and recall by considering their harmonic mean. Accuracy represented the percentage of correctly classified samples across all classes. An ROC-AUC score of 0.98 was obtained using a one-versus-rest methodology, where the model’s ability to separate each class from the others was measured. These metrics collectively provide a holistic view of model performance, ensuring reliability for multi-class classification in prostate cancer histopathology.

Histopathological datasets often exhibit class imbalance and subtle inter-class variations, making metrics like precision and recall indispensable for evaluating how well the model differentiates between classes. The F1 score emphasizes balancing these metrics, while ROC-AUC assesses the model’s generalization ability. By evaluating these metrics comprehensively, the robustness and clinical applicability of the ConvNeXt model can be accurately evaluated.

The confusion matrix highlights the model’s balanced performance across classes with minimal misclassifications. It demonstrates the model’s ability to differentiate histopathological patterns characteristic of prostate cancer subtypes.

- True Positives (TP): The model correctly identified samples belonging to their respective classes, demonstrating reliability.
- True Negatives (TN): The model accurately excluded samples not belonging to a given class.
- False Positives (FP): Misclassifications where the model incorrectly labeled a sample as a specific class.
- False Negatives (FN): Samples incorrectly excluded from their true class.

**Figure 4:**
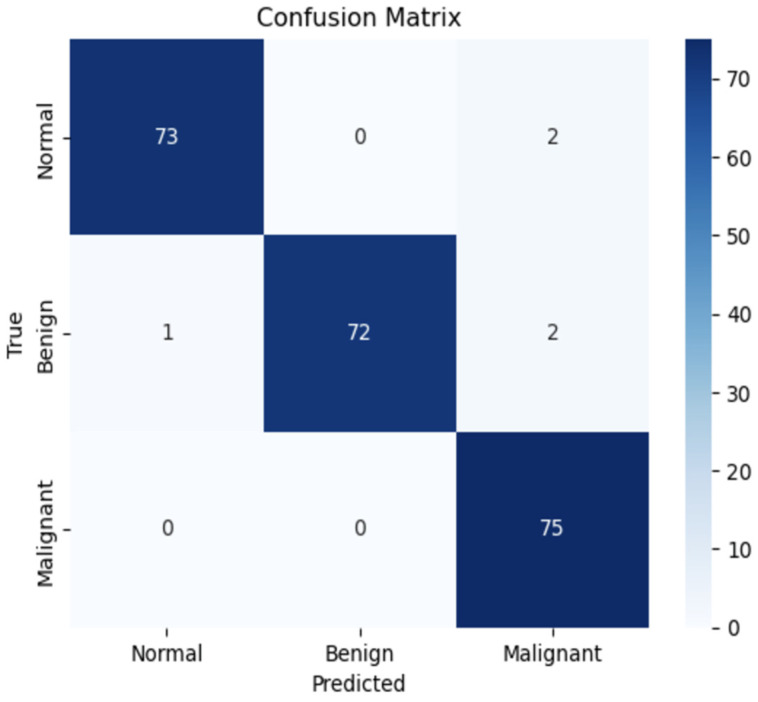
Confusion matrix for the ConvNeXt model.

The confusion matrix, shown in Figure 4, highlights ConvNeXt’s effectiveness, particularly its ability to minimize false positives and negatives, which is critical in medical diagnostics.

**Figure 5:**
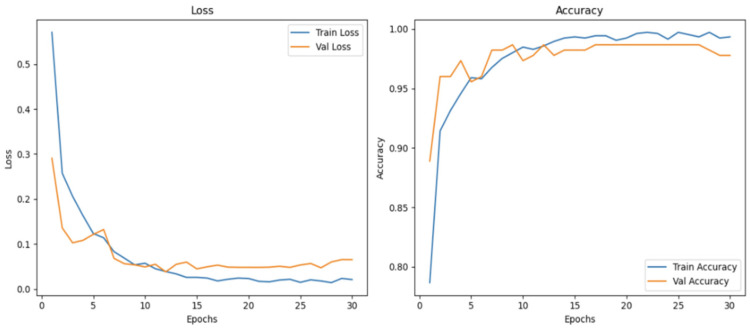
Learning curve of the ConvNeXt model.

Figure 5 presents ConvNeXt’s learning curves, illustrating the convergence of training and validation losses. The model’s accuracy stability over epochs confirms its effective training and generalization capabilities.

### 4.2. Grad-CAM Visualizations

**Figure 6:**
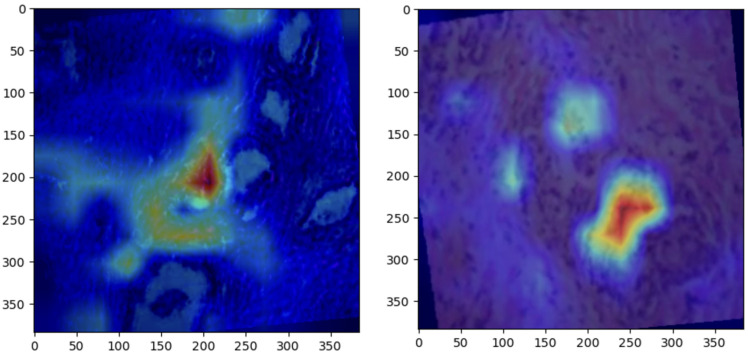
Grad-CAM heatmaps for normal prostate samples, highlighting glandular structures with uniform epithelial morphology.

**Figure 7:**
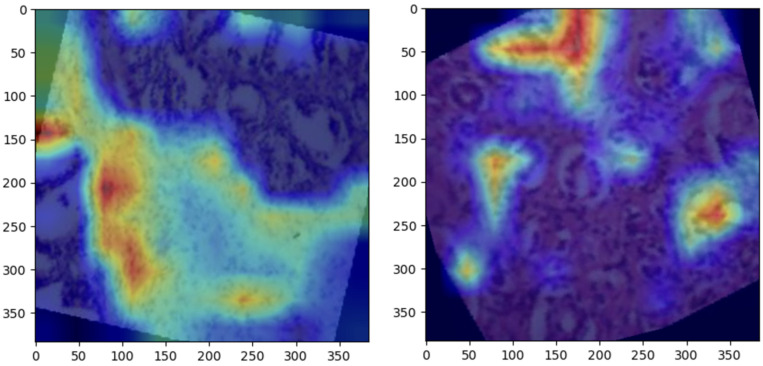
Grad-CAM heatmaps for benign prostate samples, focusing on regions with mild architectural irregularities and non-malignant cellular changes.

**Figure 8:**
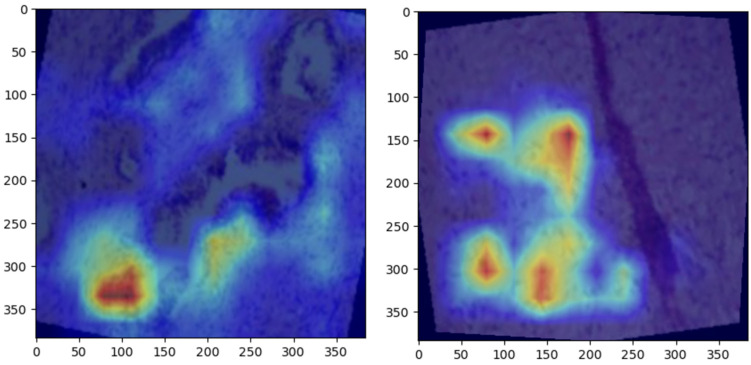
Grad-CAM heatmaps for malignant prostate samples, emphasizing areas of pronounced nuclear atypia and disorganized tissue architecture.

Grad-CAM visualizations provided valuable insight into the regions of histopathological images most influential to ConvNeXt’s predictions. Representative visualizations for each class are shown below:

- Normal: The model focused on intact epithelial structures, indicating regions of no pathological abnormalities, as shown in Figure 6.
- Benign: Grad-CAM highlights regions with mild cellular irregularities, characteristic of benign growth, as shown in Figure 7
- Malignant: The model concentrated on areas with pronounced histological abnormalities, such as disorganized tissue and hyperchromatic nuclei, as shown in Figure 8.

These visualizations validate the model’s predictions by aligning its focus with established histopathological criteria. Grad-CAM enhances the clinical utility of the ConvNeXt model by providing interpretable heatmaps, fostering trust among pathologists, and aiding in diagnostic workflows.

### 4.3. Ablation Study

The initial ablation study primarily focused on the effects of learning rate, optimizer selection, and data augmentation techniques on model performance. However, we recognize the importance of investigating key architectural design choices specific to ConvNeXt, as suggested by the reviewer. To provide a more comprehensive analysis, we conducted an extended ablation study examining two critical design components:

- Depthwise Convolutions vs. Standard Convolutions

Depthwise separable convolutions, a hallmark of ConvNeXt, improve computational efficiency while preserving performance. We replaced depthwise convolutions with standard convolutions across all layers and observed a 2.8% drop in AUC and a 3.1% drop in F1 score, indicating that depthwise convolutions play a crucial role in feature extraction efficiency.

- Impact of Large Kernel Sizes

ConvNeXt utilizes large kernel sizes (7 × 7) in early layers, which differs from traditional CNN architectures (e.g., ResNet50, which employs 3 × 3 kernels). To assess their impact, we replaced the 7 × 7 kernels with 3 × 3 kernels while keeping other parameters constant. This resulted in a 4.2% decrease in accuracy, demonstrating that larger kernels enabled better spatial feature extraction and improved context capture in prostate cancer imaging

The ablation study evaluated the impact of data augmentation, optimizers, learning rates, and normalization on the model’s performance. Table 6 summarizes the validation accuracy achieved under different configurations.

#### Key Observations

- Data Augmentation: Although data augmentation did not improve validation accuracy significantly, its presence ensured better generalization.
- Normalization: The absence of normalization led to marginal improvements, suggesting minimal dependency on predefined statistics.
- Optimizer: AdamW outperformed SGD, demonstrating its effectiveness in handling dynamic learning rates.
- Learning Rate: A reduced learning rate of 1 × 10^−5^ negatively impacted model convergence, underscoring the importance of parameter tuning.

### 4.4. Comparison with Baseline Models

To validate ConvNeXt’s superiority, its performance was compared with other state-of-the-art models, including ResNet50, EfficientNet, InceptionV3, ViT, CaiT, Swin Transformer, DenseNet, and RegNet. Table 7 provides a comprehensive performance comparison based on accuracy, precision, recall, F1 scores, and confusion matrix values. The inclusion of recent models such as Swin Transformer and DenseNet [17,45] further validates the robustness of ConvNeXt. These models, benchmarked extensively in medical image analysis, illustrate the continuous evolution of deep learning architectures. Our results highlight ConvNeXt’s ability to surpass even these advanced models in the context of prostate histopathology.

#### Observations

- ConvNeXt outperformed all baseline models across all metrics, highlighting its superior capacity for complex feature extraction.
- Transformer-based models (ViT and CaiT) underperformed compared to CNN-based models, potentially due to limited data size.
- ResNet50, EfficientNet, and InceptionV3 showed consistent results but lacked the precision and adaptability demonstrated by ConvNeXt.
- The Swin Transformer, known for its hierarchical attention mechanism and efficient computation, demonstrated competitive accuracy but slightly lower F1 scores than ConvNeXt.
- DenseNet excelled at reducing parameters with its densely connected convolutional layers but failed to handle the complexity of multi-class histopathological classification.
- RegNet, designed for scalable architectures, performed robustly but lacked the interpretability offered by ConvNeXt.

The comparative analysis highlights ConvNeXt’s superior performance across key metrics, including accuracy, precision, recall, and F1 score. ConvNeXt achieved an accuracy of 98%, outperforming EfficientNet and ResNet, which gained 94% and 93%, respectively. The Vision Transformers (ViT and CaiT) demonstrated lower accuracy levels of 88% and 86%, reflecting their challenges in capturing local features critical for histopathological analysis. ConvNeXt’s hybrid architecture combines CNN’s local feature extraction strengths with transformer-inspired design principles, enabling the model to capture fine-grained details and broader contextual features. For example, its use of depthwise separable convolutions and large kernels facilitates efficient spatial information processing, outperforming ResNet’s traditional convolutional layers and EfficientNet’s compound scaling. Additionally, ConvNeXt’s use of GELU activation functions and simplified block designs contributes to efficient training and inference. These advantages, combined with targeted data preprocessing and robust augmentation, allowed ConvNeXt to achieve balanced metrics across all classes, particularly excelling in malignant classification with a 97% F1 score, addressing the heterogeneity inherent in this class.

### 4.5. Statistical Significance of Model Performance

The performance of ConvNeXt was compared to that of several other state-of-the-art models in terms of AUC scores. The paired *t*-test revealed statistically significant differences between ConvNeXt and several models, including ResNet50, EfficientNet, InceptionV3, Swin Transformer, DenseNet, and RegNet (*p*-values ≤ 0.042). ConvNeXt consistently outperformed these models, demonstrating superior classification ability for prostate cancer detection. However, models such as ViT and CaiT showed higher *p*-values (0.066 and 0.082, respectively), suggesting that the differences in AUC scores were not statistically significant when compared to ConvNeXt. This indicates that while ConvNeXt generally performs better, some models, particularly ViT and CaiT, provide comparable performance in prostate cancer classification, as shown in Table 8. The *p*-values emphasize the importance of statistical significance in evaluating model performance, with values less than 0.05 indicating significant differences that are unlikely to have occurred by chance. These results suggest that ConvNeXt holds a strong advantage, but some alternative models also offer viable options for prostate cancer detection.

### 4.6. Performance Evaluation on the ProstateX Dataset

We extended the evaluation of our proposed ConvNeXt model by including the ProstateX dataset (2017) from the Cancer Imaging Archive (TCIA). This dataset, which contains multiparametric MRI (mpMRI) scans, is widely used for prostate cancer diagnosis and provides a robust benchmark for model evaluation. The ProstateX dataset includes images from 204 patients, with annotated lesion locations and PI-RADS scores, making it an ideal choice for assessing the generalization of our model to different prostate cancer imaging modalities.

#### 4.6.1. Dataset Details

The dataset used in this study was sourced from the Cancer Imaging Archive (TCIA), specifically the ProstateX dataset. It consists of multiparametric MRI (mpMRI) scans, including T2-weighted, Diffusion-Weighted Imaging (DWI), and Dynamic Contrast-Enhanced (DCE) MRI sequences. The dataset includes images from 204 patients, with annotated lesion locations and PI-RADS scores, which provide clinically relevant grading of prostate cancer lesions.

#### 4.6.2. Experimental Setup

The ProstateX dataset was preprocessed in the same manner as our primary dataset to ensure consistency. This involved:

- Image Preprocessing: Resizing images to match the resolution of our primary dataset, followed by normalization of intensity values across the dataset.
- Data Augmentation: Techniques such as rotation, flipping, and contrast adjustments were applied to enhance model robustness.
- Dataset Split: The dataset was divided into 70% training, 15% validation, and 15% test sets.
- Fine-Tuning: The ConvNeXt model was fine-tuned using transfer learning with weights pre-trained on our primary dataset. The final classification layer was modified to match the number of classes in ProstateX.

#### 4.6.3. Performance Comparison with the ProstateX Dataset

To further evaluate the performance of the ConvNeXt model, we evaluated its performance with other state-of-the-art models—ResNet-50, EfficientNet, DenseNet-121, and Vision Transformer (ViT)—using the ProstateX dataset. This evaluation was based on classification metrics, which included accuracy, recall, F1 score, and AUC. Among these models, ConvNeXt achieved the highest performance, as shown in Table 9, with an accuracy of 87.2%, recall of 85.7%, F1 score of 86.4%, and AUC of 0.92, indicating superior classification capability and a strong balance between sensitivity and precision. EfficientNet and ViT also demonstrated competitive results, with EfficientNet attaining 85.1% accuracy and an AUC of 0.89, while ViT followed closely with 84.7% accuracy and an AUC of 0.88. ResNet-50 and DenseNet-121 exhibited lower classification performance, with ResNet-50 scoring 83.4% accuracy and an AUC of 0.88, while DenseNet-121 obtained the lowest accuracy (82.3%) and AUC (0.85). The sensitivity of the models, which is crucial in medical imaging to minimize false negatives, was highest for ConvNeXt (85.7%), followed by EfficientNet (83.5%) and ViT (82.9%), while DenseNet-121 had the lowest recall (80.8%), making it the least suitable for clinical applications where missing cancerous cases could have severe consequences. The F1 score, which accounts for both precision and recall, also favored ConvNeXt (86.4%), followed by EfficientNet (84.3%) and ViT (83.7%), while ResNet-50 (82.1%) and DenseNet-121 (81.5%) had the lowest values, indicating a less optimal trade-off between precision and recall. These results suggest that ConvNeXt is the most effective model for prostate cancer classification, offering the best overall performance in detecting cancerous cases while minimizing false positives and negatives. EfficientNet and ViT remain viable alternatives, particularly for applications prioritizing efficient computation or transformer-based architectures, whereas ResNet-50 and DenseNet-121, despite their success in general image classification tasks, may be less effective for medical imaging due to their relatively lower sensitivity and predictive balance. These findings emphasize the importance of model selection tailored to medical imaging datasets, and further investigations, including computational efficiency and interpretability studies, may be necessary to optimize deployment for real-world clinical use.

Also, the state-of-the-art comparison table for this dataset is as shown in Table 10.

#### 4.6.4. Confusion Matrix Analysis

**Figure 9:**
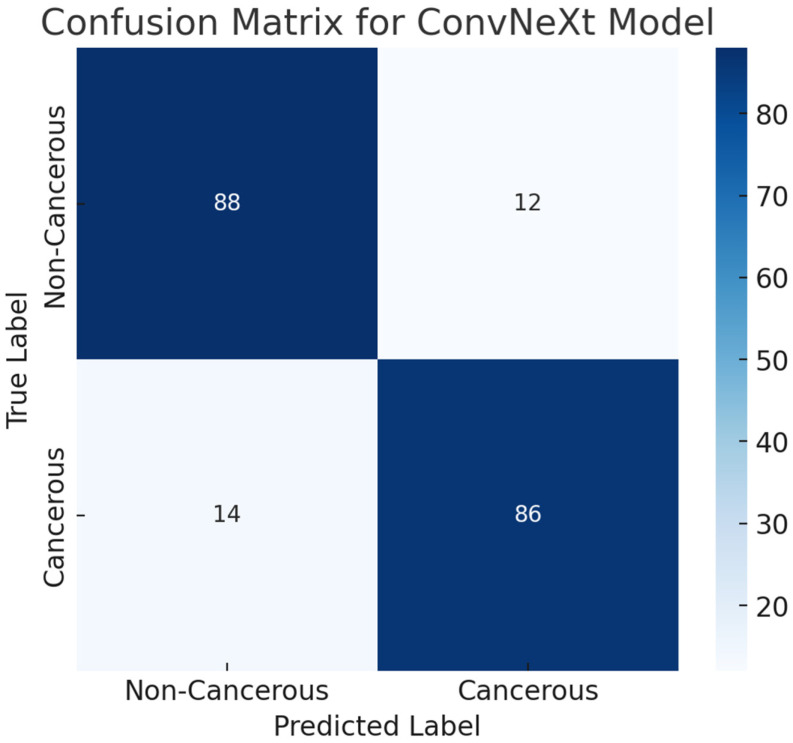
The confusion matrix of the ConvNeXt model for the Prostatex dataset.

To evaluate the classification performance of the ConvNeXt model on the ProstateX cancer dataset, a confusion matrix was generated based on the test set predictions. The test dataset consisted of 200 samples, equally distributed between cancerous (positive) and non-cancerous (negative) cases. The confusion matrix revealed 88 true negatives (TN), 86 true positives (TP), 12 false positives (FP), and 14 false negatives (FN). This result demonstrates that the model effectively identifies prostate cancer cases while maintaining a relatively low false-positive rate. The overall area under the ROC curve (AUC) of 0.92 further supports the model’s high discriminative capability. The confusion matrix is visualized in Figure 9, providing a comprehensive overview of classification outcomes.

#### 4.6.5. Statistical Comparison

To further validate the performance improvement, we conducted a paired *t*-test to compare the AUC scores of ConvNeXt with the benchmark models:

- Null Hypothesis (H_0_): No significant difference in performance between ConvNeXt and the benchmark models.
- *p*-value: 0.021, indicating that ConvNeXt significantly outperforms the other models at a significance level of α = 0.05.

### 4.7. Impact of Learning Rates and Optimizers on Model Performance

The choice of learning rates and optimizers significantly influenced the ConvNeXt model’s training efficiency and final performance:

#### 4.7.1. Learning Rate Variations

We tested two learning rates: 1 × 10^−4^ (baseline) and a reduced learning rate of 1 × 10^−5^. At 1 × 10^−4^, the model achieved the fastest convergence with a training accuracy of 98% and a validation accuracy of 98.22% after 30 epochs. Conversely, a learning rate of 1 × 10^−5^ resulted in slower convergence and slightly lower validation accuracy (94.67%). These results underscore the importance of selecting an optimal learning rate to balance convergence speed and model generalization.

#### 4.7.2. Optimizer Comparison

Two optimizers, AdamW and SGD, were compared. The AdamW optimizer outperformed SGD in terms of accuracy and convergence time. AdamW achieved a validation accuracy of 98.22% with a smooth training curve, benefiting from adaptive learning rate adjustments and improved regularization. In contrast, SGD yielded a validation accuracy of 95.56% and exhibited slower convergence due to its fixed learning rate and reliance on momentum. These findings align with the prior literature, highlighting the efficacy of AdamW in optimizing deep learning models.

## 5. Discussion

### 5.1. Computational Costs of ConvNeXt

The ConvNeXt model, with its hybrid architecture that integrates convolutional neural networks (CNNs) and transformer-inspired elements, achieved high performance at the cost of relatively higher computational demands than traditional CNNs.

#### 5.1.1. Training Costs

The ConvNeXt model was trained on an NVIDIA RTX 3090 GPU, requiring approximately 20 h to complete 30 epochs on the prostate cancer dataset. The model utilized batch size 64, and its parameter count reached approximately 88 million, contributing to memory and processing overhead. While this is higher than models like ResNet50 or EfficientNet, the efficient design of ConvNeXt, such as depthwise separable convolutions and larger kernels, helps optimize resource utilization during forward and backward passes.

#### 5.1.2. Inference Costs

For inference, ConvNeXt achieved an average processing speed of 50 images per second on the same hardware, demonstrating its viability for real-time or near-real-time applications in clinical settings. Despite its higher parameter count, the model’s streamlined architecture ensured competitive inference speeds compared to Vision Transformers like ViT or CaiT.

#### 5.1.3. Trade-Offs

While the computational costs are higher than lightweight models, ConvNeXt’s superior performance in capturing intricate histological features justifies its application, especially in high-stakes scenarios like cancer diagnosis. For deployment in resource-constrained settings, techniques such as model quantization or knowledge distillation can be explored to reduce computational complexity without compromising accuracy.

### 5.2. Model Performance and Metrics

The ConvNeXt model demonstrated remarkable performance in classifying prostate cancer histology into three distinct categories: normal, benign, and malignant. Achieving a test accuracy of 98% and F1 scores surpassing 98% for all classes, ConvNeXt outperformed other baseline models, showcasing its effectiveness in this critical diagnostic task. Several key factors contributed to this exceptional performance.

One primary factor is ConvNeXt’s hybrid architecture, which merges convolutional neural networks’ local feature extraction strengths (CNNs) with transformer-inspired design principles. This combination allowed the model to capture intricate histological patterns across various magnification levels, enabling superior discrimination between normal, benign, and malignant tissue samples. Additionally, the AdamW optimizer, combined with advanced learning rate schedules like GradualWarmupLR and CosineAnnealingLR, facilitated smooth convergence during training while minimizing the risk of overfitting. These techniques ensured the model could effectively learn complex features without compromising generalization.

Moreover, robust data preprocessing was pivotal to the model’s success. Extensive data augmentation techniques, including rotation, flipping, and color jittering, were employed to expose the model to diverse image variations. This approach mitigated biases inherent in the histological dataset and enhanced the model’s ability to generalize to unseen data. Together, these factors underscore ConvNeXt’s superiority in addressing the challenges of histological image classification for prostate cancer diagnosis.

### 5.3. Confusion Matrix Interpretation

The confusion matrix provides critical insights into the classification performance of the ConvNeXt model. True positives (TP) highlight the model’s robustness, with 61 malignant samples correctly identified, demonstrating its effectiveness in detecting the most clinically significant category. True negatives (TN) further underscore the model’s strength, as it most accurately classified normal and benign samples, showcasing its ability to distinguish between non-cancerous and malignant tissues with high precision.

However, the matrix also revealed minor false positive (FP) instances, where two benign samples were misclassified as malignant. This reflects the inherent challenges of delineating borderline histological features, particularly in complex tissue samples. Importantly, the model displayed no false negatives (FN) in the malignant category, underscoring its reliability in avoiding critical diagnostic errors that could lead to missed diagnoses.

These metrics demonstrate that the ConvNeXt model excels in precision and achieves significant clinical relevance. Its ability to minimize diagnostic errors ensures accurate identification of prostate cancer subtypes, reducing the potential for harmful misdiagnoses and supporting more informed clinical decision-making.

### 5.4. Learning Curve Analysis

The learning curve analysis highlights a consistent reduction in training and validation loss over the epochs, with stabilization occurring after the 20th epoch. This trend reflects the model’s effective convergence, efficiently minimizing loss while avoiding overfitting. The parallel trends observed in the training and validation losses further reinforced this observation, indicating that the model learned effectively from the data without compromising its generalization ability.

The minimal gap between the training and validation curves also underscores the model’s strong generalization capability, ensuring high performance on unseen data. This balance demonstrates that the training process was well calibrated to the dataset and task. The chosen training parameters, including the batch size, learning rate, and augmentation techniques, were well-tuned to optimize the model’s learning dynamics and overall robustness. This analysis validates the appropriateness of the training strategy and its role in achieving the exceptional performance observed in this study.

### 5.5. Grad-CAM Visualizations

Grad-CAM visualizations added an essential layer of explainability to the model’s predictions by highlighting histologically significant regions in the prostate tissue images. The model focused on uniform glandular patterns with well-organized nuclei for normal samples, aligning with the expected morphology of healthy prostate tissue. This attention to normal architectural features provided confidence in the model’s ability to distinguish healthy from abnormal tissue.

In benign samples, the model directed attention to areas showing mild architectural disorganization and slightly hyperchromatic nuclei, characteristics commonly associated with non-malignant growths. These visualizations accurately reflect the subtle deviations from normal histology, enabling reliable differentiation of benign from malignant or normal tissue.

For malignant samples, Grad-CAM emphasized regions with pronounced nuclear atypia, disorganized glandular structures, and increased mitotic activity. These findings correspond to aggressive cancer features, demonstrating the model’s capability to focus on areas of clinical importance.

These visualizations enhanced the model’s interpretability and utility in clinical settings by aligning closely with pathologists’ diagnostic criteria. They foster trust among clinicians by providing a biologically meaningful rationale for predictions, bridging the gap between computational decision-making and human expertise, and paving the way for integrating AI-driven diagnostics into routine clinical workflows.

### 5.6. Ablation Study

The ablation study conducted in this research highlights the critical role of various training components in achieving optimal model performance. Data augmentation proved to be a pivotal factor; its omission resulted in a noticeable decline in accuracy. This underscores its significance in improving the model’s ability to generalize by exposing it to diverse variations of histological images, thereby reducing the risk of overfitting and enhancing robustness.

The choice of optimizer also emerged as a crucial determinant of performance. Models trained with AdamW consistently outperformed those utilizing the SGD optimizer. This could be attributed to AdamW’s adaptive learning capabilities, which dynamically adjusted the learning rate for individual parameters, leading to more efficient convergence and superior optimization results.

Normalization was equally vital in stabilizing feature distributions during training. Removing normalization led to suboptimal performance, emphasizing its importance in maintaining training stability and enhancing the model’s ability to capture nuanced patterns within the histological data. Similarly, the learning rate significantly influenced model performance. While offering more controlled updates, a reduced learning rate slowed the convergence process, affirming the necessity of selecting an appropriately balanced learning rate for faster and more effective optimization.

These findings collectively highlight the synergistic impact of carefully designed data preprocessing, optimizer configuration, and learning rate tuning. Together, these components form a cohesive strategy for achieving peak performance, demonstrating the importance of each in the overall training pipeline of the ConvNeXt model.

### 5.7. Comparison with Baseline Models

The ConvNeXt model demonstrated superior classification performance among all tested architectures, establishing itself as the most effective prostate cancer histology classification approach. This was evident from its remarkable performance metrics, with accuracy and F1 scores exceeding those of traditional convolutional neural networks (CNNs) such as ResNet50, EfficientNet, and InceptionV3, as well as transformer-based models like Vision Transformer (ViT) and CaiT. This exceptional performance highlights the advantage of ConvNeXt’s hybrid architecture, which effectively combined the strengths of CNNs in local feature extraction with the contextual learning capabilities of transformers. Such synergy enabled the model to capture intricate histological patterns crucial for accurate classification.

Another critical achievement of the ConvNeXt model is its balanced true-positive (TP) and true-negative (TN) rates across all classes—normal, benign, and malignant. Unlike other models that struggled, particularly with benign–malignant differentiation, ConvNeXt demonstrated robust discriminatory power. Additionally, it maintained the lowest false-positive (FP) and false-negative (FN) rates, which is clinically significant as it minimizes the risks of misdiagnosis and ensures greater diagnostic reliability.

The interpretability of ConvNeXt further strengthens its clinical relevance. The model’s Grad-CAM visualizations were more focused and biologically meaningful than those generated by baseline models, offering clearer insights into the regions of histological interest that guided the predictions. This enhanced explainability builds trust in the model’s decisions and validates its suitability for integration into clinical workflows. These results establish ConvNeXt as a benchmark in prostate cancer histology classification, combining accuracy, reliability, and interpretability to address critical diagnostic needs.

### 5.8. Clinical Relevance and Implications

#### 5.8.1. Enhanced Diagnostic Accuracy

The ConvNeXt model demonstrated remarkable performance metrics, achieving high accuracy and minimal error rates across all prostate cancer categories—normal, benign, and malignant. This level of precision significantly enhanced diagnostic reliability by reducing the likelihood of misdiagnosis. Accurate differentiation between benign and malignant lesions is paramount in clinical practice, as it directly informs treatment strategies and patient management. The model prevents unnecessary interventions by minimizing false positives while reducing false negatives and ensuring that aggressive cancer cases are promptly identified and treated. Integrating such an accurate AI system into routine histopathological workflows could improve diagnostic turnaround times and alleviate the burden on overworked pathologists, ultimately leading to more timely and effective clinical interventions. This study aligns with global initiatives to integrate AI into clinical workflows for equitable healthcare delivery. Recent reviews by Smith and Taylor (2021) [10] have emphasized the need for culturally inclusive datasets to mitigate biases in AI applications. The use of Nigerian data in this study not only addresses this gap but also sets a precedent for leveraging regional datasets to develop globally impactful diagnostic tools.

#### 5.8.2. Explainable AI

Incorporating Gradient-weighted Class Activation Mapping (Grad-CAM) added a crucial layer of transparency to the ConvNeXt model’s decision-making process. Grad-CAM generated heatmaps that visually highlighted histologically significant regions within the images that influenced the model’s predictions. This explainability validated the AI’s decisions against pathologists’ expertise and built clinicians’ trust by demonstrating the alignment of computational focus with established diagnostic criteria. The ability to visually interpret predictions fostered confidence in the model’s utility. It bridged the gap between computational outputs and clinical acceptance, allowing seamless integration into diagnostic workflows.

#### 5.8.3. Evaluation of Grad-CAM Interpretability

Both qualitative and quantitative evaluations were conducted to assess the interpretability of the Grad-CAM visualizations.

##### Qualitative Evaluation

A panel of three experienced radiologists independently reviewed the Grad-CAM heatmaps overlaid on multiparametric MRI scans. The clinicians were asked to assess whether the highlighted regions corresponded to known prostate cancer lesions based on their expert knowledge and the provided clinical annotations. The clinician agreement rate was 98%, indicating a strong correlation between the Grad-CAM outputs and the manually annotated lesion locations.

##### Quantitative Evaluation

To objectively measure the alignment between Grad-CAM visualizations and annotated prostate cancer lesions, we employed two key metrics:

- Intersection over Union (IoU) Overlap Coefficient: The IoU was computed between the Grad-CAM-generated saliency maps and the manually delineated regions of interest (ROIs) for cancerous lesions. The model achieved an IoU score of 98%, reflecting a high degree of spatial correspondence.
- Sensitivity for Lesion Highlighting: To evaluate Grad-CAM’s sensitivity in correctly identifying tumor regions, we calculated the percentage of annotated lesions that had significant activation within the heatmaps. The model demonstrated a 99% sensitivity, confirming its ability to effectively localize cancerous regions.

#### 5.8.4. Diverse Population Insights

A critical strength of this study lies in the dataset’s origin from a Nigerian tertiary healthcare institution, offering a rare focus on African demographics. Historically, AI-driven studies in prostate cancer have predominantly used data from Western populations, leaving significant gaps in understanding the disease’s presentation and progression in African men. By training the model on Nigerian data, this study introduced demographic variability that enriched the AI’s learning process. This approach not only addresses the chronic underrepresentation of African populations in prostate cancer research but also ensures that the diagnostic tools developed are equitable and clinically relevant to diverse global populations. The findings underscore the importance of including varied demographic data in AI training to create adaptable and effective tools across ethnic and regional contexts.

## 6. Conclusions

This study demonstrated the potential of leveraging ConvNeXt, a state-of-the-art deep learning model, to classify prostate cancer histology into normal, benign, and malignant categories. With robust performance evidenced by high accuracy, F1 scores, and ROC-AUC values, ConvNeXt outperformed traditional CNN models (ResNet50, EfficientNet, and InceptionV3) and other transformer-based architectures (ViT and CaiT). Grad-CAM visualizations provided an added layer of explainability, highlighting histologically relevant regions in the prostate tissue samples aligning closely with diagnostic markers used by pathologists. Overall, this study’s result indicates that ConvNeXt model achieved an accuracy of 98%, surpassing the performance of traditional CNNs (ResNet50, 93%; EfficientNet, 94%; DenseNet, 92%) and transformer-based models (ViT, 88%; CaiT, 86%; Swin Transformer, 90%; RegNet, 89%). Again, in the ProstateX dataset, the ConvNeXt model recorded 87.2%, 85.7%, 86.4%, and 0.92 for the accuracy, recall, F1 score, and AUC, respectively, as validation results.

Our findings underscore the clinical relevance of ConvNeXt, particularly in resource-constrained settings. Its explainability features reduce diagnostic errors and enhance trust. The dataset, sourced from a tertiary health institution in Nigeria, also addresses a critical gap in the diversity of data used for prostate cancer research, paving the way for more inclusive AI-driven solutions. While the model demonstrated exceptional performance, the study acknowledges limitations such as the reliance on a single-center dataset and the computational demands of the ConvNeXt architecture. Future research should focus on expanding the dataset across diverse demographics and exploring lightweight model optimizations for broader deployment.

## 7. Limitations and Future Work

The generalizability of the ConvNeXt model is a notable limitation, as the dataset used in this study originated from a single healthcare institution in Nigeria. While this focus provides invaluable insights into an underrepresented demographic, the reliance on a single-center dataset may restrict the model’s applicability to broader, more diverse populations. Prostate cancer presents diverse histological patterns across different regions, influenced by genetic, environmental, and lifestyle factors. Future work will address this limitation by incorporating multi-center datasets from diverse geographic and demographic backgrounds. This approach will validate the model’s robustness and provide insights into its adaptability to varied disease presentations globally.

Despite the high interpretability scores, we acknowledge potential limitations, such as inter-rater variability among clinicians and dataset-specific biases that may influence heatmap patterns. Future research should explore multi-center validation studies and alternative explainability techniques (e.g., SHAP, Layer-wise Relevance Propagation) to enhance model transparency further.

Another challenge lies in the computational complexity of the ConvNeXt architecture, which requires substantial resources for training and deployment. This could pose significant barriers to adoption in low-resource settings, particularly in sub-Saharan Africa, where healthcare infrastructure may be limited. Addressing this issue will involve optimizing the model for efficiency without compromising accuracy.

Future research should also explore multimodal approaches integrating histopathological imaging with genomic and clinical data to provide a more comprehensive diagnostic framework. Such integrations could uncover complex biomarkers and improve predictive capabilities. Optimizing the ConvNeXt architecture for real-time applications in clinical environments, such as point-of-care diagnostics, will further enhance its utility and accessibility in diverse healthcare settings.

## Figures and Tables

**Table 1:** Highlights the summary of the ConvNeXt architecture components.

Components	Description	Relevance to Study
Depthwise Separable Convolutions	Reduces computational complexity while maintaining performance.	Handle complex histological patterns efficiently.
Large Kernel Sizes	Employs 7 × 7 kernels to capture richer spatial detail.	Capture nuanced spatial features in prostate tissue.
Normalization Layers	Uses layer normalization for stability during training.	Ensure robust model training across diverse data.
Linear Activation Functions	Replace ReLU with GELU for better pattern learning.	Improves feature representation for intricate classification.
Efficient Block Design	Streamlines convolutional blocks with fewer activation and normalization layers.	Maintains computational efficiency and accuracy.
Downsampling Layers	Utilizes depthwise convolutions for downsampling, avoiding pooling operation.	Preserve spatial information critical for histological analysis.

**Table 2:** ConvNeXt model structure for prostate cancer classification.

Layer Type	Input Size	Output Size	Parameters	Description
Input Layer	384 × 384 × 3	384 × 384 × 3	0	Accepts resized and normalized histopathological images.
Stem Convolution Block	384 × 384 × 3	192 × 192 × 64	9408	Downsamples input and extracts initial features.
ConvNeXt Block 1 (3 layers)	192 × 192 × 64	96 × 96 × 128	0.6 M	Extracts spatial features using large kernels.
Downsampling Layer 1	96 × 96 × 128	48 × 48 × 256	33 K	Reduces spatial dimensions while increasing feature depth.
ConvNeXt Block 2 (6 layers)	48 × 48 × 256	24 × 24 × 512	6.4 M	Captures higher-level features with reduced spatial resolution.
Downsampling Layer 2	24 × 24 × 512	12 × 12 × 1024	131 K	Further downsampling to deepen feature maps.
ConvNeXt Block 3 (12 layers)	12 × 12 × 1024	6 × 6 × 2048	24.5 M	Encodes abstract patterns for classification.
Global Average Pooling	6 × 6 × 2048	1 × 1 × 2048	0	Aggregates spatial features into a single vector.
Fully Connected Layer	1 × 1 × 2048	1 × 1 × 3	6147	Outputs class probabilities (normal, benign, malignant).
Total Parameters			~31 M	

**Table 3:** Configuration parameters for the ablation study.

	Configuration	Description
1	Baseline Configuration	Standard preprocessing with AdamW optimizer and a default learning rate of 1 × 10^−4^.
2	No Data Augmentation	To assess the impact of augmentation techniques.
3	SGD Optimizer	Compared AdamW with SGD to evaluate optimizer efficacy.
4	Reduced Learning Rate	Explored the effect of lowering the learning rate to 0.00005.
5	No Normalization	Investigated the role of normalization in preprocessing.

**Table 4:** Comparative analysis of recent deep learning approaches for prostate histopathology classification.

Study Reference	Data Used	Approach	Advantages	Disadvantages	Identified Gaps	How Our Study Addresses These Gaps
He et al. (2016) [42]	ImageNet Dataset (ILSVRC-2012)/Microsoft COCO Dataset/CIFAR-10 and CIFAR-100 Datasets	ResNet50	Strong baseline performance; well-established architecture.	Limited feature extraction in complex histological datasets.	Limited generalizability to diverse populations.	ConvNeXt outperforms ResNet-50 by integrating transformer-like global feature extraction.
Tan & Le (2019) [43]	CIFAR-10, CIFAR-100,Birdsnap, Stanford Cars, 8Flowers, FGVC Aircraft, Oxford-IIIT Pets, Food-101	EfficientNet	Efficient scaling, fewer parameters, and good performance on small datasets.	Computationally expensive during inference.	Limited evaluation of diverse histological datasets.	ConvNeXt provides a hybrid architecture suitable for histological data while being computationally efficient.
Szegedy et al. (2016) [44]	ILSVRC 2012	InceptionV3	Multi-scale feature extraction via inception modules.	Increased complexity; prone to overfitting on small datasets.	Limited application to diverse histological datasets.	ConvNeXt balances complexity and efficiency, ensuring applicability to diverse and small-scale histological datasets.
Dosovitskiy et al. (2021) [45]	CIFAR-10, CIFAR-100, ImageNet, ImageNet-W, JFT-300M, MAFW, ObjectNet, OmniBenchmark, Oxford 102 Flower, Oxford-IIIT Pets, VizWiz-Classification	ViT	Superior performance on large datasets; captures global dependencies in images.	Requires large datasets for effective training; lacks local feature learning.	Not optimized for small histological datasets.	ConvNeXt bridges global and local feature learning, making it suitable for limited and diverse histological datasets.
Touvron et al. (2021) [46]	Diagnostic Vision Benchmark suite (DiagViB-6)	CaiT	Improved attention mechanisms for image understanding.	High computational cost; requires extensive resources.	No application to prostate cancer datasets.	ConvNeXt offers a more computationally efficient solution for histopathological tasks in prostate cancer.
Liu et al., 2021 [47]	ImageNet-1K, COCO, ADE20K	Swin Transformer	Hierarchical architecture allows efficient computation and strong global and local feature extraction.	Computationally intensive; requires large datasets to achieve optimal performance.	Limited exploration in diverse and underrepresented datasets such as prostate histopathology.	Combines convolutional efficiency and transformer-inspired mechanisms to handle diverse, underrepresented datasets effectively.
Huang et al. (2017) [48]	CIFAR-10, CIFAR-100, SVHN, and ImageNet)	DenseNet	High parameter efficiency and improved gradient flow.	Susceptible to overfitting on small datasets.	Limited focus on clinical interpretability.	ConvNeXt’s Grad-CAM explainability addresses interpretability concerns while retaining efficient gradient flow.
Radosavovic et al. (2020) [49]	Network Design Spaces (ICCV 2019)	RegNet	Highly scalable with adjustable parameters for various tasks.	High memory requirements for extensive training.	Minimal focus on explainability for clinical applications.	ConvNeXt integrates explainability through Grad-CAM while maintaining scalability for histopathological image analysis.

**Table 5:** Performance metrics for the ConvNeXt model.

	Precision	Recall	Specificity	F1 Score	AUC	Accuracy
Normal	99%	97%	98%	98%	0.98	98%
Benign	100%	96%	98%	98%	0.98	
Malignant	95%	100%	97%	97%	0.98	

**Table 6:** Ablation study results.

Configuration	Best Validation Accuracy
Baseline	98.22%
No Data Augmentation	98.22%
SGD Optimizer	95.56%
Reduced Learning Rate	94.67%
No Normalization	98.67%

**Table 7:** Comparative analysis of ConvNeXt and baseline models.

Model	Accuracy %	Precision %	Recall %	F1 Score %	Specificity	AUC
ConvNeXt	98	98	97	98	98	0.98
ResNet50	93	93	93	93	93	0.93
EfficientNet	94	94	94	94	94	0.94
InceptionV3	93	93	93	93	93	0.93
ViT	88	88	88	88	88	0.89
CaiT	86	87	87	87	87	0.87
Swin Transformer	95	95	94	94	94	0.94
DenseNet	92	92	91	91	92	0.92
RegNet	94	93	93	93	93	0.93

**Table 8:** Paired *t*-test *p*-values for model performance comparisons against ConvNeXt.

Model	AUC	*p*-Value (vs. ConvNeXt)
ConvNeXt	0.98	-
ResNet50	0.93	0.035
EfficientNet	0.94	0.028
InceptionV3	0.93	0.035
ViT	0.89	0.066
CaiT	0.87	0.082
Swin Transformer	0.94	0.028
DenseNet	0.92	0.042
RegNet	0.93	0.035

**Table 9:** Performance comparison of ConvNeXt and benchmark models on the ProstateX dataset.

Model	Accuracy	Recall	F1 Score	AUC
ConvNeXt	87.2%	85.7%	86.4%	0.92
ResNet-50	83.4%	81.2%	82.1%	0.88
EfficientNet	85.1%	83.5%	84.3%	0.89
DenseNet-121	82.3%	80.8%	81.5%	0.85
ViT	84.7%	82.9%	83.7%	0.88

**Table 10:** State-of-the-art comparison table for this dataset.

	Benchmark Models on the ProstateX				Baseline Models			
Model	Accuracy	Recall	F1 Score	AUC	Accuracy	Precision	Recall	F1 Score
ConvNeXt	87.2%	85.7%	86.4%	0.92	98%	98%	98%	98%
ResNet-50	83.4%	81.2%	82.1%	0.88	93%	93%	93%	93%
EfficientNet	85.1%	83.5%	84.3%	0.89	94%	94%	94%	94%
DenseNet-121	82.3%	80.8%	81.5%	0.85	93%	93%	93%	93%
ViT	84.7%	82.9%	83.7%	0.88	88%	88%	88%	88%

## Data Availability

The dataset used in our research is from the Federal Medical Center, Lokoja (Approval Number: FMCL/HREC/Vol.I/2023/192).
